# EMDL_m6Am: identifying N6,2′-O-dimethyladenosine sites based on stacking ensemble deep learning

**DOI:** 10.1186/s12859-023-05543-2

**Published:** 2023-10-25

**Authors:** Jianhua Jia, Zhangying Wei, Mingwei Sun

**Affiliations:** School of Information Engineering, Jingdezhen Ceramic University, Jingdezhen, 333403 China

**Keywords:** m^6^Am site identification, Stacking, Deep learning, DenseNet, DCNN, MSRN

## Abstract

**Background:**

N6, 2'-O-dimethyladenosine (m^6^Am) is an abundant RNA methylation modification on vertebrate mRNAs and is present in the transcription initiation region of mRNAs. It has recently been experimentally shown to be associated with several human disorders, including obesity genes, and stomach cancer, among others. As a result, N6,2′-O-dimethyladenosine (m^6^Am) site will play a crucial part in the regulation of RNA if it can be correctly identified.

**Results:**

This study proposes a novel deep learning-based m^6^Am prediction model, EMDL_m6Am, which employs one-hot encoding to expressthe feature map of the RNA sequence and recognizes m^6^Am sites by integrating different CNN models via stacking. Including DenseNet, Inflated Convolutional Network (DCNN) and Deep Multiscale Residual Network (MSRN), the sensitivity (Sn), specificity (Sp), accuracy (ACC), Mathews correlation coefficient (MCC) and area under the curve (AUC) of our model on the training data set reach 86.62%, 88.94%, 87.78%, 0.7590 and 0.8778, respectively, and the prediction results on the independent test set are as high as 82.25%, 79.72%, 80.98%, 0.6199, and 0.8211.

**Conclusions:**

In conclusion, the experimental results demonstrated that EMDL_m6Am greatly improved the predictive performance of the m^6^Am sites and could provide a valuable reference for the next part of the study. The source code and experimental data are available at: https://github.com/13133989982/EMDL-m6Am.

## Introduction

In recent years, dynamic epigenetic changes of RNA have drawn considerable attention in biological study. There are already more than 160 different forms of RNA modifications known [1]. According to reports, the majority of RNA modification enzyme mutations have an important role in the emergence of human disorders [2]. In rat messenger RNA (mRNA), the N6-methyladenosine (m^6^A) modifications were discovered [3], while N6-2′-O-methyladenosine (m^6^Am) alterations were discovered shortly [4]. One of the most prevalent post-transcriptional modifications of mRNA is N6,2′-O-methyladenosine (m^6^Am), which was found on the second base of the modification close to the m7G cap [4]. In the meantime, m^6^Am, a terminal alteration generally 2′-O-methylated at the second base close to the 50 cap in mRNA, together with further methylation at the N6 position, has just recently been Identified as a promising target for FTO removal from the human obesity gene [5], associated with obesity [6].

Through the experimental studies, researchers have been able to uncover more and more hidden characteristics of m^6^Am. Recent research has gradually revealed the importance of m^6^Am in biological functions, including in the field of cardiac biology [7] research, gene expression regulation [8], tumor development, and more [9]. For example, PCIF1 and m^6^Am were experimentally proven to be essential in the development of gastric cancer by Zhuo et al. [10]. The progression of gastric cancer is accelerated by increased methyltransferases driven by m^6^Am methylation changes (PCIF1). By providing resistance to DCP2-mediated mRNA capping, M^6^Am confers mRNA [11]. Furthermore, m^6^Am may also control other processes involving the metabolism of RNA. The experimental findings of Jan Mauer et al. [11] demonstrated that m^6^Am plays a significant role in the stability [11–13] and translation [14] function of mRNA. Overall, research on m^6^Am's biological effects is still in its early stages, and its primary functions are yet unknown. There are numerous wet-lab experimentation techniques available. As an illustration, Hawley et al. [15] developed miCLIP, a mapping of m^6^A and m^6^Am at single nucleotide precision, to find the m^6^Am sites. Additionally, m6ACE-seq was introduced by Koh et al. [16] to statistically map m^6^A and m^6^Am across the transcriptome. There is also the MeRIP-seq (m^6^A-seq), which has limited resolution, antibody cross-reactivity, and inability to differentiate between cap-m^6^Am and m^6^A in 50 RNA fragments.

Many other researchers are still striving to find m^6^Am sites using wet experimental approaches nowadays, including Sun et al. [17], they used antibodies to m^6^Am to recognize m^6^Am, but there was a limitation that they cannot precisely distinguish between m^6^Am and 5′-UTR m^6^A. Because the existing wet experimental techniques are expensive and time-consuming, it is vital to develop new computational methods for the exact identification of m^6^Am sites.

Despite the fact that prediction of m^6^Am sites have not been studied for a long time, a number of prediction techniques have been developed. The first one bases its prediction identification on conventional machine learning model. Based on RNA sequence and employing electron–ion interaction and pseudo-EIIP (PseEIIP) coding to predict sites, Jiang et al. [18] developed a predictor called m6AmPred, and the team continued to identify m^6^Am sites computationally using deep learning algorithms with proposing a new predictor called MultiRM [19]. As deep learning framework, based on the LSTM-attention mechanism combined with Word2vec embedding module, can perform multi-tag prediction, including prediction of m^6^A, m^6^Am, and other 12 RNA modification sites with powerful features.

Recently, Luo et al. [20] used a different coding approach and model than Jiang to predict m^6^Am. They used three coding approaches—one-hot coding, nucleotide chemical properties, and nucleotide density—as well as three base classifiers—multi-headed attention, two parallel embedding modules, CNN and BiLSTM, and a prediction module for m^6^Am sites. As a whole, the computational identification of m^6^Am sites has made some strides, that the algorithms now in use can predict m^6^Am sites with a high level of performance and accuracy. However, there is still a lot that can be done to increase the precision of these predictors for m^6^Am sites identification.

**Fig. 1:**
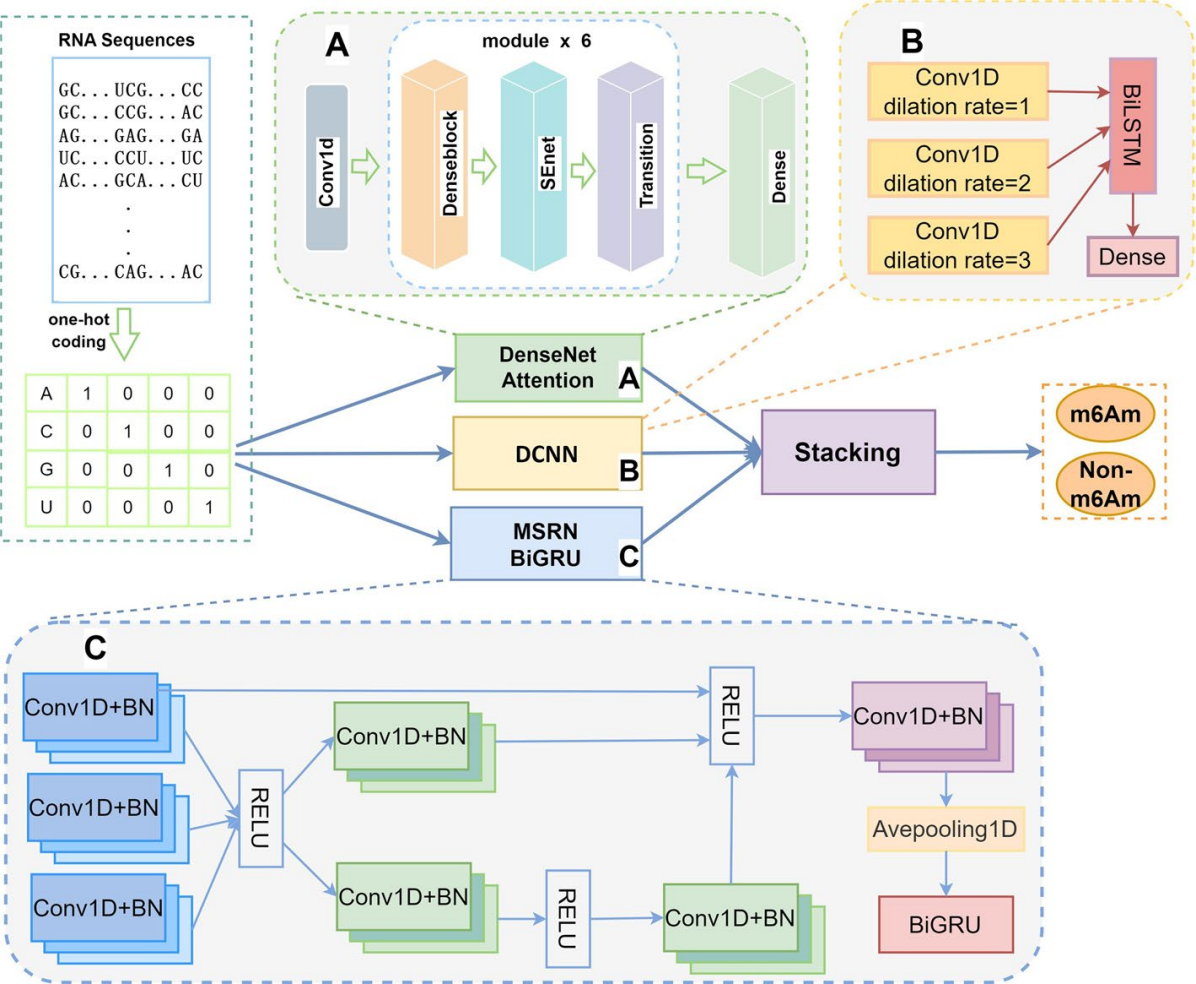
Schematic graph of the EMDL_m6Am model

Based on the above-mentioned consideration, we propose EMDL_m6Am, a stacking ensemble deep learning model, in response to the current mission of identifying m^6^Am sites. It ismotivated by those m^6^Am data and the certain sites prediction models. The whole flowchart is displayed in Fig. 1. The model consists of an encoding module for features and a feature extraction module. The raw data is initially encoded in one step. Then, three deep learning models—A, B, and C—are divided up into the feature extraction module, and stacking integration is added later. Conv1D is for a one-dimensional convolutional layer. Avepooling1D stands for an average pooling layer, a fully connected layer is denoted Dense for, and BN for batch normalization.

As shown in Fig. 1, first, we attempted to use a number of popular RNA sequence coding techniques, such as one-hot, nucleotide chemical property (NCP), and nucleotide density (ND), both singly and in combination. Through the prediction performance obtained from the experiment, we finally selected the most straightforward, effective coding technique, one-hot, for RNA sequence coding. Second, we examined the performance of different deep learning predictors, including: DenseNet [21, 22], Inflated Convolutional Network and Deep Multiscale Residual Network(MSRN) [23] and Bidirectional gating recurrent unit (BiGRU), and got some inspirations from them. Through carefully testing, we discovered that the DenseNet network based on one-hot encoding performs well. DenseNet still has drawbacks when compared with the currently state-of-the-art predictor DLm6Am, though. Hence, giving that the performance gaps amongst the three deep learning classifiers are not very large, we built a stacking ensemble deep learning model and used a straightforward logistic regression model for prediction in the second layer of stacking. Three deep learning models are employed in the first layer of stacking to forecast the original data to obtain the prediction results, and the second layer filters the first layer’s prediction outcomes. Notably, we optimized the input of the second layer of the stacking model in this work, i.e., the output values of the first layer were stitched with the original dataset to serve as the input of the second layer, in order to avoid losing the original feature map information, which was inspired by the stacking prediction model from Jia et al. [24]. Finally, the code is obtainable in the Github repository (https://github.com/13133989982/EMDL-m6Am).

## Materials and methods

### Benchmark dataset

Lately, Sun et al. [17] proposed a new sequencing approach, m6Am-seq, which can effectively distinguish m^6^Am from m^6^A using RNA immunoprecipitation and selective external demethylation. From this, they used this sequencing approach to provide 2166 m^6^Am sites in the entire human transcriptome at mononuclear resolution with a high confidence level for these sites. Subsequently, luo et al. [20] did a three-step process on the sites information provided by m6Am-seq: first, a sample sequence was extracted using a sliding window of $(2\delta + 1)-nt$*,* as shown in Eq. (1), and a sequence can be displayed as:

1 $${f}_{\delta }\left(K\right)={A}_{-\delta }{A}_{-\left(\delta -1\right)}\cdots {A}_{-2}{A}_{-1}K{A}_{+1}{A}_{+2}\cdots {A}_{+\left(\delta -1\right)}{A}_{+\delta }$$

whereas in BCA (B = C, G, or U) motifs, *K* stands for the nucleotide adenosine and *A* for the nucleotide next to *K*. The distance between each nucleotide and the central site *K* is indicated by the subscript, with *A*_(−δ) standing for the δ-th upriver nucleotide from the middle, *A*_(+δ) denoting the δ-th downriver nucleotide from the center, and so on. In this study, the δ value was configured for 20. The sequence fragment is regarded as a positive sample if the m^6^Am site is in the middle of it; otherwise, it is a negative sample. Secondly, sequences with more than 80% similarity were removed using the redundancy removal tool CD-HIT [25], Finally, negative samples were chosen at random in a 1:1 ratio to create the ultimate benchmark dataset, from which 80% of the samples were chosen as the training set and the residual 20% as the independent test set. The dataset used in our work is from the dataset created in three steps by Luo et al. and we did not do additional processing on their dataset. The size of the dataset used is shown in Table 1, the training set contains 1419 positive samples and 1419 negative samples, and the test set has 355 positive samples and 355 negative samples. The ratio of positive to negative samples is 1:1 in both sets of data.

**Table 1:** Distribution of the benchmark data set

Dataset	Positive	Negative
Training	1419	1419
Independent	355	355

### Feature extraction methods

Use of an appropriate sequence coding approach is essential for site identification. Researchers have created a number of feature encoding techniques to express RNA sequences with numerical vectors since the input of the majority of models is a numerical vector. As an illustration, we consider the encoding based on the structural or sequence information. In this research, we employ three of the most well-applied coding techniques at present, including one-hot, NCP, and ND [26, 27].

### One hot encoding

One-hot coding is one of the most prevalent coding techniques and is well-liked by scientists since it can effectively and simply represent nucleotide sequences. The four nucleotides A (adenine), C (cytosine), G (guanine), and U (uracil) are typically denoted by (1, 0, 0, 0), (0, 1, 0, 0), (0, 0, 1, 0) and (0, 0, 0, 1), accordingly.

Therefore, a nucleotide sequence of length *L* = 41 is represented in this study as a 41 × 4 two-dimensional matrix, where all matrix elements are 0 and 1.

### Nucleotide chemical property (NCP) and nucleotide density (ND))

The chemical characteristics of nucleotides (NCP), which was proposed by Bari et al. [28], based on the construction of chemical structures of nucleotides, have been used in many recent investigations [29–31].

1. The nucleotides are classified into these two categories based on the various ring configurations. As purines, A and G are represented by the number 1, whereas pyrimidines, C and U, are denoted by the number 0.
2. According to their chemical functions, nucleotides are separated into two groups: those representing amino groups, designated by the number 1, such as the nucleotides A and C, and those representing keto groups, denoted by the number 0, such as the nucleotides G and U.
3. Based on the different strengths of the hydrogen bond interactions between base pairs, the nucleotides are divided into two groups. One party comprised the strong interactions and is represented by 1, which is A and U, while the other group represents the weak interactions and is represented by 0, which is G and C.

In this way, each nucleotide is represented by the following three-dimensional vector.

2 $$Hi = \left({x}_{i}, {y}_{i}, {z}_{i}\right)$$

where $$x_{i} = \left\{ {\begin{array}{*{20}l} {1,} \hfill & {H_{i} \in \left\{ {A,G} \right\}} \hfill \\ {0,} \hfill & {H_{i} \in \left\{ {C,U} \right\}} \hfill \\ \end{array} } \right.$$ denotes the ring structures, $$y_{i} = \left\{ {\begin{array}{*{20}l} {1,} \hfill & {H_{i} \in \left\{ {A,G} \right\}} \hfill \\ {0,} \hfill & {H_{i} \in \left\{ {G,U} \right\}} \hfill \\ \end{array} } \right.$$ indicates the chemical efficiency, $$z_{i} = \left\{ {\begin{array}{*{20}l} {1,} \hfill & {H_{i} \in \left\{ {A,U} \right\}} \hfill \\ {0,} \hfill & {H_{i} \in \left\{ {C,G} \right\}} \hfill \\ \end{array} } \right.$$ represents the interaction strength of hydrogen bonds.

The frequency and location distribution information of each nucleotide can be displayed in the nucleotide density (ND). The density (*d*_*i*_) of a nucleotide can be calculated as *d*_*i*_ = *n/i*, where *n* is the number of times the nucleotide appears before the *i*-th position (including the *i*-th position). For example, the density of A for the sequence "AGTAUUCA" is 1, 0.50, and 0.375 in the first, fourth, and eighth bits, respectively. Likewise, U is 0.20, 0.33 for positions 5 and 6, respectively, etc.

Combining the chemical properties and density of nucleotides for encoding, then each nucleotide can be encoded as a four-dimensional vector shown in Eq. (3).

3 $$Ni=\left\{{x}_{i}, { y}_{i}, { z}_{i},{ d}_{i} \right\}\left(i = 1, 2, 3, \dots l\right)$$

where* l* stands for the sequence’s length. Each sequence data of length 41 nt can be encoded as a two-dimensional numerical matrix of 41 × 4.

### Classification model

It is necessary to select the right model in order to correctly anticipate the m^6^Am sites. As the main network structure in this study, three deep learning models were selected, and stacking was then employed to integrate them for site prediction.

### DenseNet and attention

#### DenseNet

**Fig. 2:**
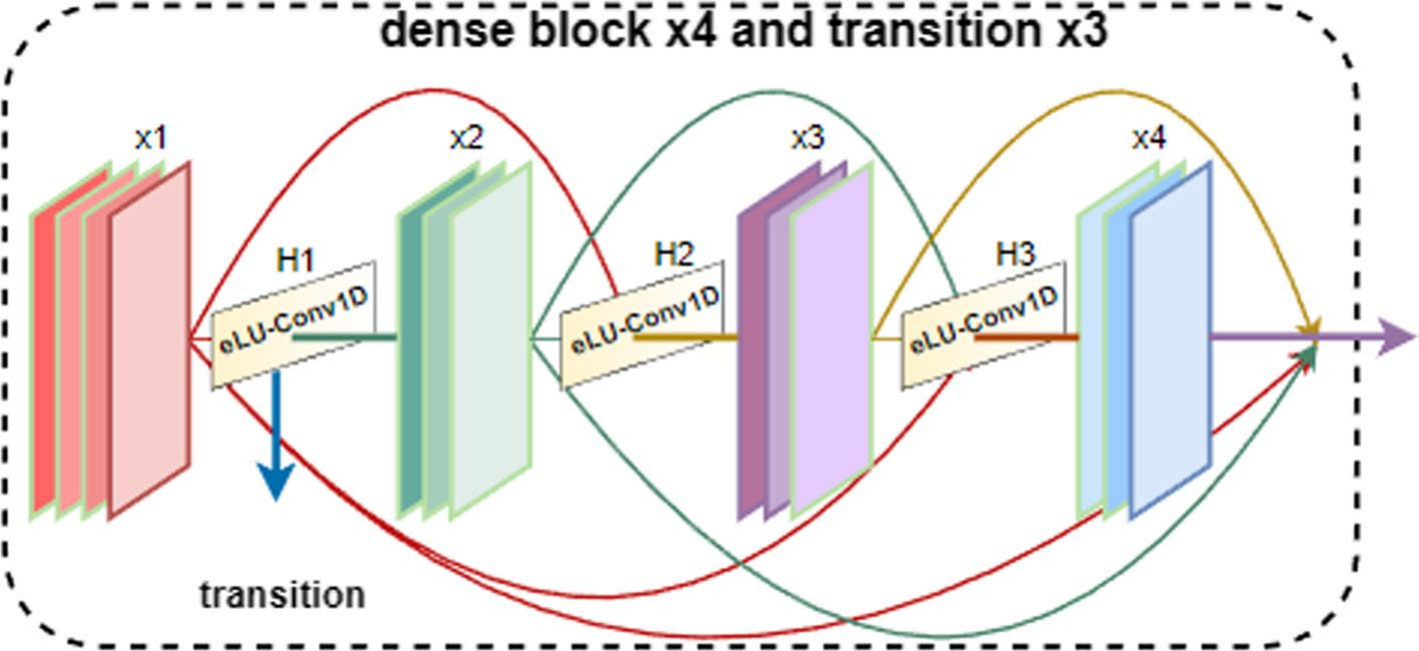
Connection of the dense convolutional layer

Considering the DenseNet [32], a dense connectivity technique, each network layer is connected to the next by a feedforward connection that transfers data across layers. In order to prevent model overfitting, the gradient disappearance problem is effectively addressed, feature propagation is enhanced, feature reuse is promoted, and the number of parameters is drastically reduced. It has been demonstrated that DenseNet outperforms conventional CNNs because it focuses on both low-level and high-level feature information based on a dense hopping connection mechanism to satisfy the goal of mutual and complementary information transfer. Figure 2 shows the connecting mechanism.

Wang et al. [21] employed DenseNet to predict Lysine Acetylation Sites and achieved great performance. Jia et al. [33] successfully implemented the lysine succinylation sites prediction in the DenseNet network in while utilizing an attention module to assess the significance of various feature information. As a result, we were motivated to use the model’s denseblock module to record feature data at various levels. The output of all the preceding layers is passed on to the next layer as input by each layer in the DenseNet. Therefore, *L*(*L* + 1)/2 connections exist across all layers of a DenseNet with *X* layers. In other words, Eq. (4) represents the output of the network’s *L*-th layer.

4 $${x}_{i}={H}_{i}\left(\left[{x}_{0}, {x}_{1},\dots ,{x}_{i-1}\right]\right)$$

where H(∙) stands for the non-linear transformation function and eLU and conv1D are included.

#### SENet

We investigated incorporating attention mechanisms into the model to help the model learn crucial feature knowledge in the principle of earlier studies [21, 34]. We investigated the individual and joint impacts of channel attention, spatial attention, and Squeeze-and-Excitation Networks (SENet). For instance, adding channel attention at the end of the dense net output and adding SENet attention after each dense block inside the DenseNet constitute the combined application of the attention mechanism. The best results, according to ablation investigations, came from applying compressed attention by itself.

Since the attention layer is directly related to the input matrix, it is important to note that we introduced it before DenseNet. This helps the model pay attention to the underlying data and supports the identification of critical location information to avoid distraction. The results of the experiment showed how valuable an attention layer like this is for improving the accuracy of model predictions.To increase the weight of important information and sharpen the attention on it, SENet is added concurrently after each denseblock to the feature map information output. The standard implementation of SENet looks like this.First, the input feature map is initially subjected to globelAvgPooling, which results in the feature map's spatial dimension being compressed. Second, to reduce dimensionality and suit the correlation between channels, a fully connected operation is performed on the compressed feature map.Next, the compressed feature map is connected in its entirety. The downscaled feature map is upscaled in the third phase such that Sigmoid can be used to obtain the normalized weights between 0 and 1.Finally, to create the final feature map weighting, the normalized weights are added to the features of each channel.

Specially, this study’s SENet is a modified version of the original SENet. In order to achieve feature map information weighting while reusing the original feature map to prevent information loss, the concept of residual network was specifically used to add the original feature map to the matrix created after compression.

### DCNN and BiLSTM

Holschneider et al. [35] initially announced dilation convolution, which added intervals to conventional convolution [36] while maintaining the resolution of the feature map. In contrast to standard convolution, dilation convolution contains an additional parameter called the dilation rate, which is primarily used to describe the size of the dilation. It is a normal convolution when the dilation rate is 1.

Wang et al. [23] developed the integrated multiscale deep learning predictor EMDLP, which combined inflated convolutional neural network (DCNN) with BiLSTM for the prediction of RNA methylation. Shortly after, Liu et al. [37] proposed MSNet-4mC, which combined residual network concepts to identify 4mC loci and used DCNN to extract feature map data at various scales. We adapted the joint application of DCNN and BiLSTM to the loci prediction of m^6^Am after being encouraged by the prior studies. Starting with sequence data, the learning function f(x) of m^6^Am divides into four segments: one-hot encoding, dilated convolution to extract features, splicing feature map, and extracting contextual information using BiLSTM as shown in Eq. (5).

5 $$\mathbf{h}=f(x)={f}_{\text{BiLSTM }}\left({f}_{\text{concat }}\left({f}_{\mathrm{DCNN}}\left({f}_{\text{one-hot-encoding }}\left(\mathbf{x}\right)\right)\right)\right)$$

The formula for the dilated convolution in a one-dimensional circumstance is given in Eq. (6). Different dilution rates can be thought of as adding different of blank gaps among each convolution kernel.

6 $${y}_{i}=f\left(\sum_{n=1}^{N} {x}_{i+k*n}{\omega }_{n}+b\right)$$

where *x*_*i*_ is the *i*-th input item, *y*_*i*_ is the *i*-th DCNN array’s output, ω is the filter’s weight, *N* is the filter’s longer, and *k* is referred to by the dilution rates (DR).

The dilution rate for each of the three blocks of DCNNs in the DCNN stage is 1, 2, and 3, the information of the feature map is learned from three different sensory fields. And, each DCNN block consists of a max-pooling layer with a dropout unit, a dilated convolutional layer with the rectified linear unit (ReLU) as its active function, and a dilated convolutional layer.

### MSRN and BiGRU

To find the circRNA-RBP interaction locations, Niu et al. [23] employed a deep multiscale residual network and a network made up of BiGRU with a self-attention mechanism. Both MSRN and BiGRU can effectively represent high-level features and are competent at learning local and global contextual information. Thus, aiming at predicting the m^6^Am sites, we attempted to cascade multiple CNNs of various scales and use them in conjunction with BiGRU. The residuals are introduced in the CNN cascade to ensure that important feature map features are not lost during the convolution phase. So as to prevent information loss, the model also includes BiGRU, which handles the long-term dependencies of the sequences and maintains important aspects utilizing a range of gating functions.

First, we extracted features from a one-hot encoded feature map using six cascaded multi-scale residual blocks (MSRBs), each of which consists of three convolutional layers and 64 convolutional kernels.The output of each MSRB is then integrated to produce global feature fusion. A further 1D convolution and average pooling procedure is then carried out with 192 convolution filters.

Second, to improve the recognition performance of the model and to make up for the fact that the multiscale residual network can only extract sequence correlation and not context-linked information, the output of the deep multiscale residual network is fed into BiGRU to obtain contextual information [38].

### Stacking ensemble

In general, a prediction task will produce the different prediction outcomes in distinct predictors. Ensemble learning can combine numerous classifiers to obtain the prediction results that are superior to those by a single classifier. Among them, stacking is a key data science technique that depends on the outcomes of numerous models. By the stacking approach, the prediction outcomes of various models are trained once again as features, and the resulting prediction consequences frequently beat those of a single strong model.

**Fig. 3:**
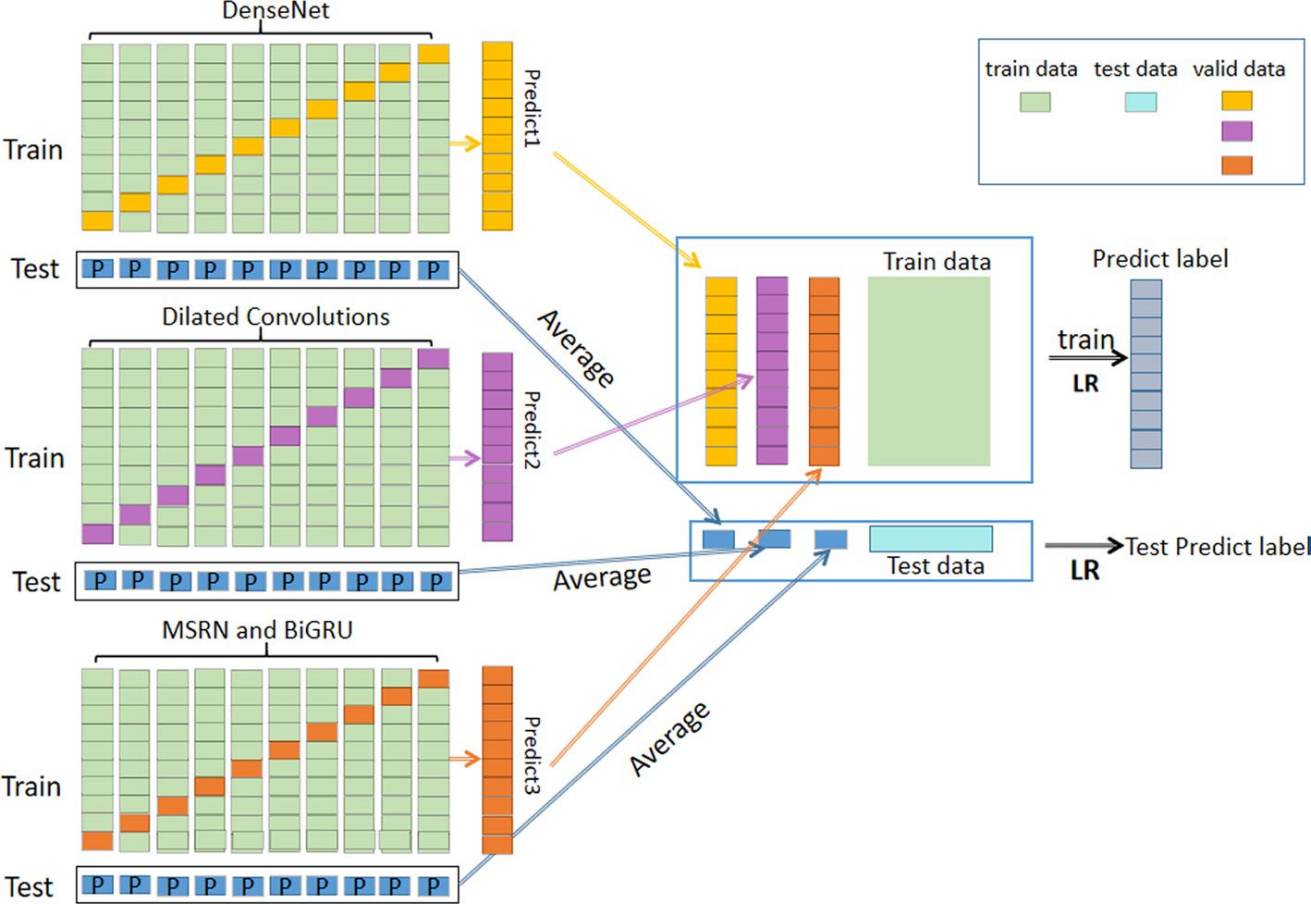
The stacking ensemble model scheme

DenseNet, DCNN, and MSRN were the three classifiers we intended to use in this study as the basic classifiers in the first layer. In particular, in order to prevent the information loss and overfitting, the outputs of the first-layer’s three classifiers were mixed with the original dataset as the input for the second-layer’s classifier. Here, the second-layer classifier that we used was a straightforward logistic regression model. Figure 3 displays the structural framework of the stacking ensemble model.

### Performance evaluation

The foundation for evaluating the effectiveness of various models is the employment of common and scientific measures. The following four metrics are commonly used to predict performance for binary classification problems [39, 40] in general machine learning, as represented by the following metric equations:

7 $$\begin{array}{*{20}l} {Sn = \frac{TP}{{TP + FN}}} \hfill \\ {Sp = \frac{TN}{{TN + FP}}} \hfill \\ {Acc = \frac{TP + TN}{{TP + TN + FP + FN}}} \hfill \\ {MCC = \frac{TP \times TN - FP \times FN}{{\sqrt {\left( {TP + FN} \right) \times \left( {TN + FN} \right) \times \left( {TP + FP} \right) \times \left( {TN + FP} \right)} }}} \hfill \\ \end{array}$$

where respectively, TP, TN, FP, and FN stand for the quantity of incorrect predictions in the positive classification, the quantity of incorrect predictions in the negative classification, the number of accurate predictions in the positive category, and the number of correct predictions in the negative category. In addition, the model's efficiency is often evaluated using the area under the receiver operating characteristic curve (AUROC) and the area under the precision and recall curves (AUPR).

## Result and discussion

### Contrasting various feature extraction techniques

Regarding our experiments in the feature extraction section, we use the three feature encoding techniques one-hot, NCP, and ND. The majority of studies [15, 18, 20] use NCP in conjunction with ND. There are three possible combinations in this manner: one-hot alone, NCP and ND combined, and one-hot and NCP, ND combined. As indicated in Tables 2 and 3, we compare the performance of different feature extraction approaches by performing fivefold cross-validation on the training set and independent tests on the test set, where the best predicted results are shown in bold.

**Table 2:** Ablation studies of the feature encoding ways on train set

Encoding methods	Sn ± SD (%)	Sp ± SD (%)	ACC ± SD (%)	MCC ± SD	AUC ± SD	AUR ± SD
One hot	**86.62 ± 0.11**	**88.94 ± 0.09**	**87.78 ± 0.09**	**0.7590 ± 0.1725**	**0.8778 ± 0.0878**	**0.8428 ± 0.1078**
NCP and ND	84.64 ± 0.08	84.29 ± 0.07	84.47 ± 0.07	0.6908 ± 0.1294	0.8447 ± 0.0654	0.7948 ± 0.0756
One-hot, NCP and ND	84.08 ± 0.07	85.06 ± 0.07	84.57 ± 0.06	0.6928 ± 0.1209	0.8457 ± 0.0609	0.7977 ± 0.0738

The best experimental results are shown in bold

**Table 3:** Ablation studies of the feature encoding ways on test set

Encoding methods	Sn (%)	Sp (%)	ACC (%)	MCC	AUC	AUR
One hot	**82.25**	**79.72**	**80.98**	**0.6199**	0.8211	0.7626
NCP and ND	81.13	78.31	79.72	0.5946	0.8191	0.7621
One-hot, NCP and ND	80.28	79.44	79.86	0.5972	**0.8230**	**0.7716**

The best experimental results are shown in bold

We used one-hot coding as the only coding method for RNA sequence data as the model input data, because Tables 2 and 3 show that when one-hot coding alone was used as the feature coding method, and the important evaluation indexes like Sn, Sp, ACC in cross-validation and independent testing were higher than other individual or combined coding methods.

### Comparison of models with different denseblocks

**Fig. 4:**
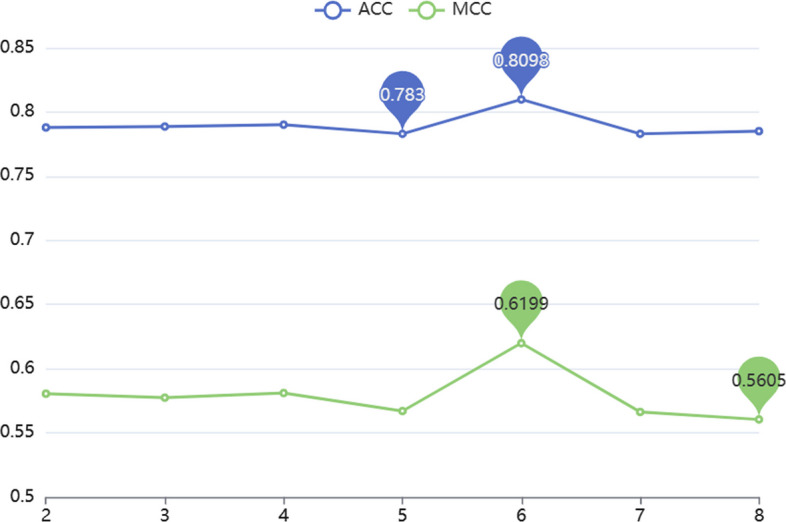
Comparison of ACC, MCC values for predictors with different number of denseblocksin DenseNet

The parameters of the predictor are optimized in DenseNet by setting different numbers of denseblocksand the performance of the model is compared by ACC and MCC values. It can be clearly seen in Fig. 4 that the predictor performs best when DenseNet picks 6denseblocks.

### Model architecture ablation experiment

**Fig. 5:**
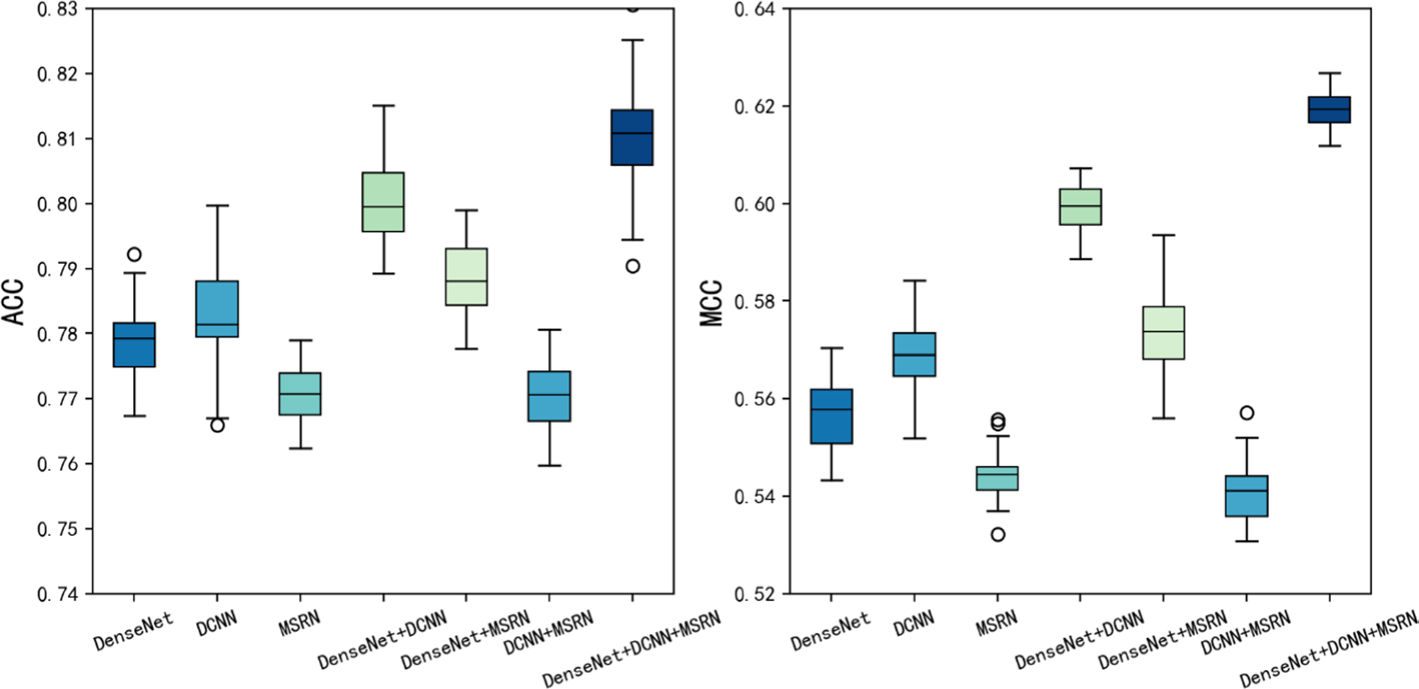
Performance evaluation of seven classifiers with three different model combinations through test sets

When choosing models, ablation studies were carried out to evaluate which of the three models—or which conjunction of the three—performed better. Table 4 and Fig. 5 display the predictions made by the three deep learning models alone and in conjunction. Similar to Table 4, the mark "√" in the row of every deep learning model denotes the model's selection for this study; meanwhile, the absence of mark "√" (i.e. blank)—denotes the unselecting of the prediction model.

**Table 4:** Ablation studies of the models on train set

DenseNet	√			√	√		√
DCNN		√		√		√	√
MSRN			√		√	√	√
Sn ± SD (%)	77.10 ± 0.08	83.38 ± 0.09	75.61 ± 0.06	85.35 ± 0.10	85.63 ± 0.07	83.09 ± 0.10	**86.62 ± 0.11**
Sp ± SD (%)	75.33 ± 0.07	79.14 ± 0.08	75.54 ± 0.06	86.26 ± 0.07	86.69 ± 0.06	82.17 ± 0.07	**88.94 ± 0.09**
ACC ± SD (%)	76.22 ± 0.02	81.26 ± 0.07	75.58 ± 0.02	85.81 ± 0.07	86.16 ± 0.06	82.63 ± 0.06	**87.78 ± 0.09**
MCC ± SD (%)	**76.29 ± 0.05**	62.74 ± 0.15	75.88 ± 0.04	71.73 ± 0.15	72.47 ± 0.12	65.64 ± 0.12	75.90 ± 0.17
AUC ± SD (%)	76.30 ± 0.03	88.89 ± 0.07	75.50 ± 0.03	85.81 ± 0.08	86.16 ± 0.06	82.63 ± 0.06	**89.78 ± 0.09**
AUR ± SD (%)	52.99 ± 0.05	**88.88 ± 0.07**	51.49 ± 0.05	81.29 ± 0.09	81.62 ± 0.07	77.22 ± 0.07	84.28 ± 0.11

The best experimental results are shown in bold

Table 4 and Fig. 5 clearly show that when employing stacking to combine the three deep models DenseNet, DCNN, and MSRN, ACC and MCC for the model were greater than those of the other cases on the train and test sets. Therefore, to extract features from the original data and forecast the m^6^Am sites in this study, we integrated DenseNet, DCNN, and MSRN using stacking.

### Comparative analysis of other models

**Fig. 6:**
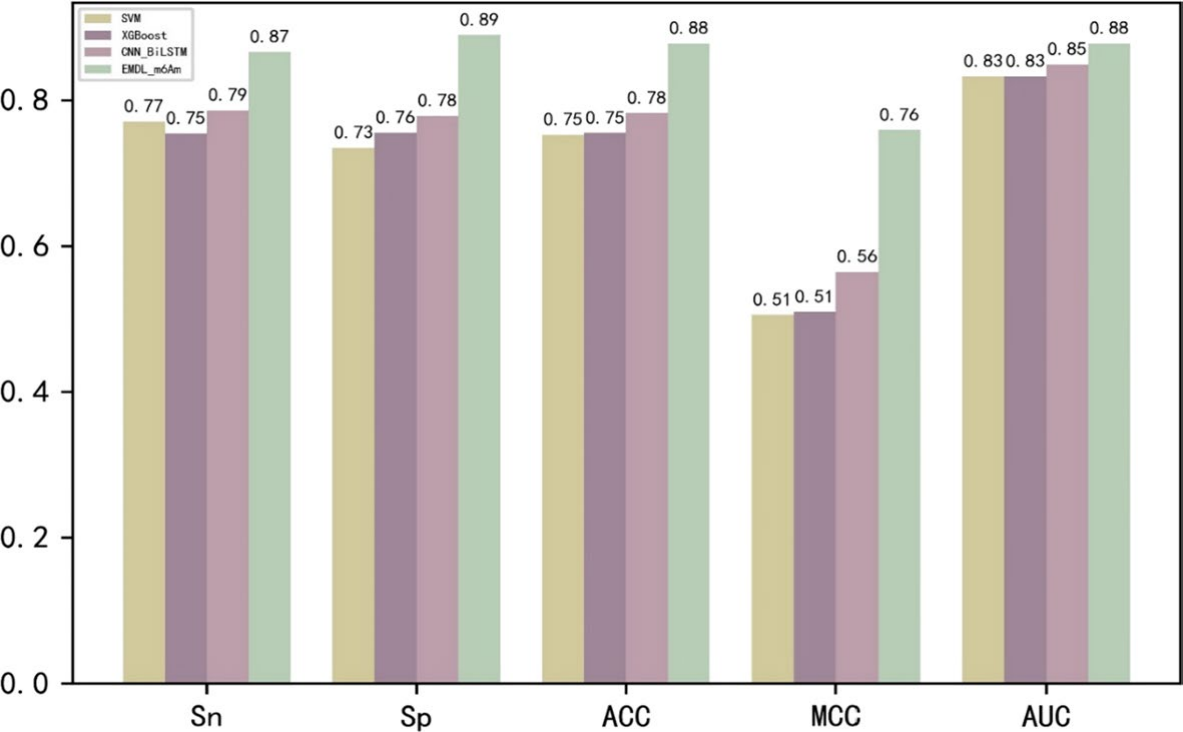
Comparison of performance between different models on training data

We compared the performance of EMDL_m6Am with a number of representative models, including the conventional machine learning model SVM, XGBoost and the deep learning model CNN and BiLSTM. The results of the fivefold cross-validation of various models on the training set were shown in Fig. 6. This demonstrated that the deep learning model outperformed machine learning in the extraction of features from huge datasets. Additionally, it was clear that EMDL_m6Am exceeds other models in terms of accuracy when predicting the m^6^Am sites.

**Fig. 7:**
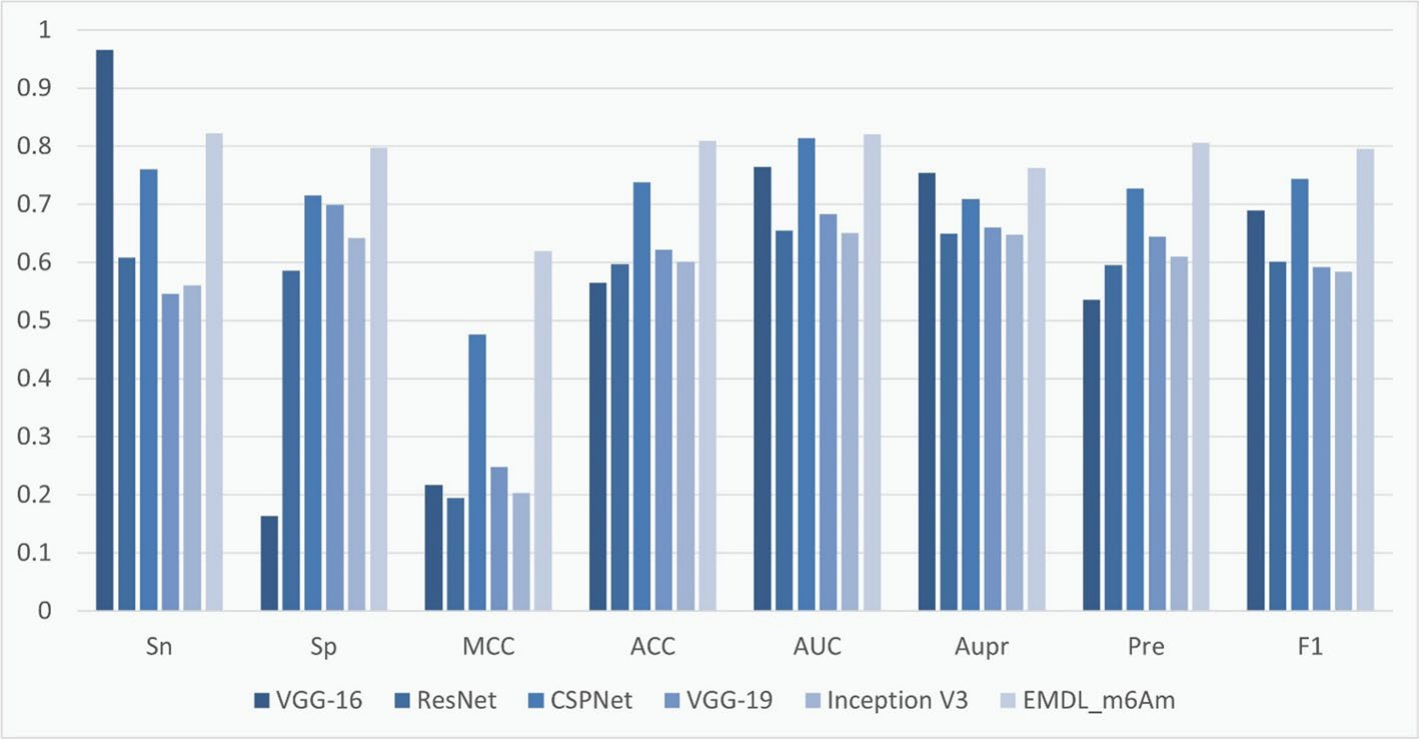
Comparison of the prediction performance of EMDL_m6Am and five advanced models on an independent test setComparison of different classifiers

To assess the prediction performance of EMDL_m6Am, we alse compared EMDL_m6Am with several advanced models for analysis, including: Cross Stage Partial DenseNet (CSPNet) [41], VGG-16 [42], ResNet [43], VGG-19 [44], Inception V3. These models performed differently on the independent test set, as shown in Table 5. Among them, VGG-16 scored the highest in Sn, but the Sp score was too low, lost the balance between Sn and Sp, and had too much deviation for high prediction accuracy. In contrast, our model EMDL_m6Am achieved a balance between Sn and Sp with a deviation of less than 2.25%, and obtained the highest Sp, MCC, Acc, AUC, AUPR, Pre, and F1 scores among several models, yielding the best prediction results overall. To clearly demonstrate the superiority of EMDL_m6Am, we plotted Fig. 7 to show the performance of several models.

**Table 5:** Performance of several advanced models on independent test sets

model	Sn	Sp	MCC	ACC	AUC	AUPR	Pre	F1
VGG-16	**0.9662**	0.1634	0.2173	0.5648	0.7643	0.7546	0.5359	0.6894
ResNet	0.6085	0.5859	0.1944	0.5972	0.6549	0.6500	0.5951	0.6017
CSPNet	0.7606	0.7155	0.4765	0.7380	0.8135	0.7094	0.7278	0.7438
VGG-19	0.5465	0.6986	0.2480	0.6225	0.6830	0.6604	0.6445	0.5915
Inception V3	0.5606	0.6423	0.2035	0.6014	0.6510	0.6482	0.6104	0.5844
EMDL_m6Am	0.8225	**0.7972**	**0.6199**	**0.8098**	**0.8211**	**0.7626**	**0.8061**	**0.7960**

The best outcomes are in bold

**Fig. 8:**
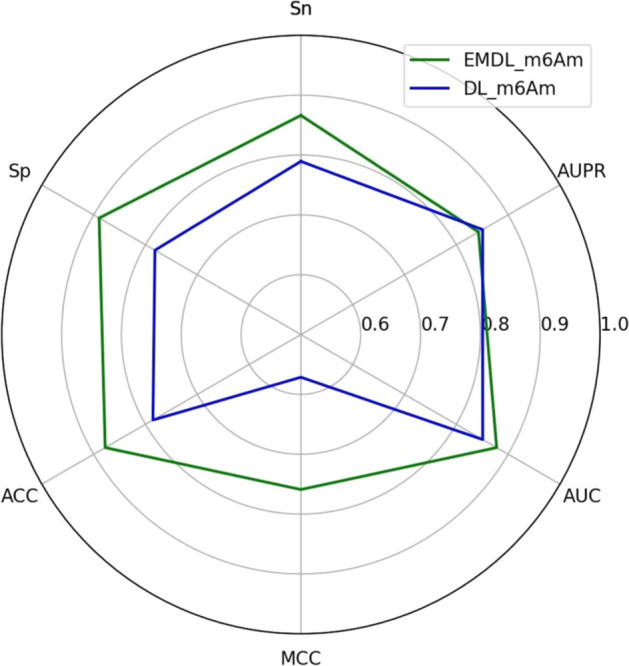
Comparison of EMDL_m6Am and DLm6Am on test Sets

We even farther confirmed the effectiveness of EMDL_m6Am by contrasting it with other present predictors for predicting m^6^Am sites in RNA sequences, including MultiRM, m6AmPred, and DLm6Am. The four models all used the same dataset to ensure the validity of the experimental comparison. Tables 6 and 7 presented the findings of the fivefold cross-validation of the four models on the training and test sets. It was evident that EMDL_m6Am had significantly better prediction performance on the training set than the other three models, and that it had 7% more Sn, 10% more Sp, 7%–8% more ACC, and 7%–8% more MCC than the most recent model, DLm6Am. When used to balanced datasets, AUPR is less accurate and is less effective and informative for dichotomizing since it is susceptible to sample distribution. Table 7 showed that EMDL_m6Am performed better than the other three models on the independent test set, which also indicated that EMDL_m6Am had good generalization ability. A direct viewing comparison of the two predictors was shown in Fig. 8. This demonstrated that EMDL_m6Am outperformed the current state-of-the-art model DLm6Am.

**Table 6:** The fivefold cross-validation performance of different predictors

Predictor	Sn	Sp	ACC	MCC	AUC	AUPR
MultiRM*	0.7378	0.6751	0.7065	0.4100	0.7827	0.7777
m6AmPred*	0.7378	0.7209	0.7294	0.4588	0.8085	0.8105
DLm6Am*	0.7921	0.7893	0.7907	0.5814	0.8545	**0.8532**
EMDL_m6Am	**0.8662**	**0.8894**	**0.8778**	**0.7590**	**0.8778**	0.8428

The conclusions were from the previous study, as stated by the asterisk (*) [20]

The best experimental results are shown in bold

**Table 7:** Comparison of different predictors on test dataset

Predictor	Sn	Sp	ACC	MCC	AUC	AUPR
MultiRM*	0.7859	0.6366	0.7113	0.4273	0.8058	0.7977
m6AmPred*	0.7211	0.7408	0.7310	0.4621	0.8205	0.8208
DLm6Am*	0.8171	0.7740	0.7955	0.5916	**0.8633**	**0.8634**
EMDL_m6Am	**0.8225**	**0.7972**	**0.8098**	**0.6199**	0.8230	0.7716

The conclusions were from the research, as stated by the asterisk (*) [20]

The best experimental results are shown in bold

To test the robustness of the model and to evaluate the performance of the model on unbalanced datasets, we used the full transcript dataset and the mature RNA dataset from m6AmPred [18] on m^6^Am. It is worth noting that we used the original unbalanced dataset, in which the full transcript dataset contains 2447 positive samples and the mature RNA dataset includes 1673 positive samples, with the ratio of positive to negative samples being 1:4. The full transcript dataset and the mature RNA dataset were randomly divided into a training set and a test set in the ratio of 8:2. Our model EMDL_m6Am without any non-equilibrium processing on the dataset, only used the network framework structure of the model for feature extraction and weight assignment to get the final prediction results. Finally, the comparison of the results of EMDL_m6Am and m6AmPred on the two datasets was shown in Table 8.

**Table 8:** Performance comparison of m6AmPred and EMDL_m6Am on independent test sets

Dataset	Methods	Sn	Sp	ACC	MCC
Full transcript	m6AmPred	0.5460	**0.9865**	0.9464	0.6352
	EMDL_m6Am	**0.6401**	0.9771	**0.9568**	**0.6577**
Mature RNA	m6AmPred	0.3791	**0.985057**	0.9299	0.4895
	EMDL_m6Am	**0.4567**	0.9798	**0.9371**	**0.4940**

The best outcomes are in bold

As shown in Table 8, on the full transcript dataset, the Sn, ACC and MCC values of EMDL_m6Am were higher than those of m6AmPred, and only the Sp values were all slightly lower than it. The same was true on the mature RNA dataset. Collectively, our model EMDL_m6Am outperformed m6AmPred, and also illustrated that EMDL_m6Am performed equally well on the unbalanced dataset with good generalization ability.

**Fig. 9:**
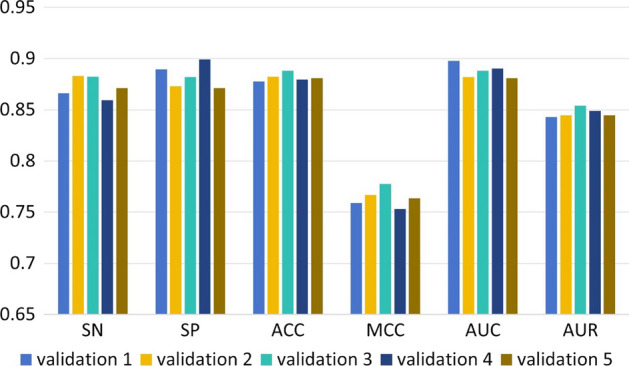
Results of five tenfold cross-validation

In order to verify the robustness of EMDL_m6Am, we randomly selected negative samples several times to conduct five tenfold cross-validation experiments. The results of the experiments were shown in Fig. 9, and the results of the five tenfold cross-validation experiments were similar without too much difference. This indicated that EMDL_m6Am had stable experimental results for selecting different sets of negative samples.

### Sequence analysis of m^6^Am sites and T‑SNE visualization of EMDL_m6Am

**Fig. 10:**
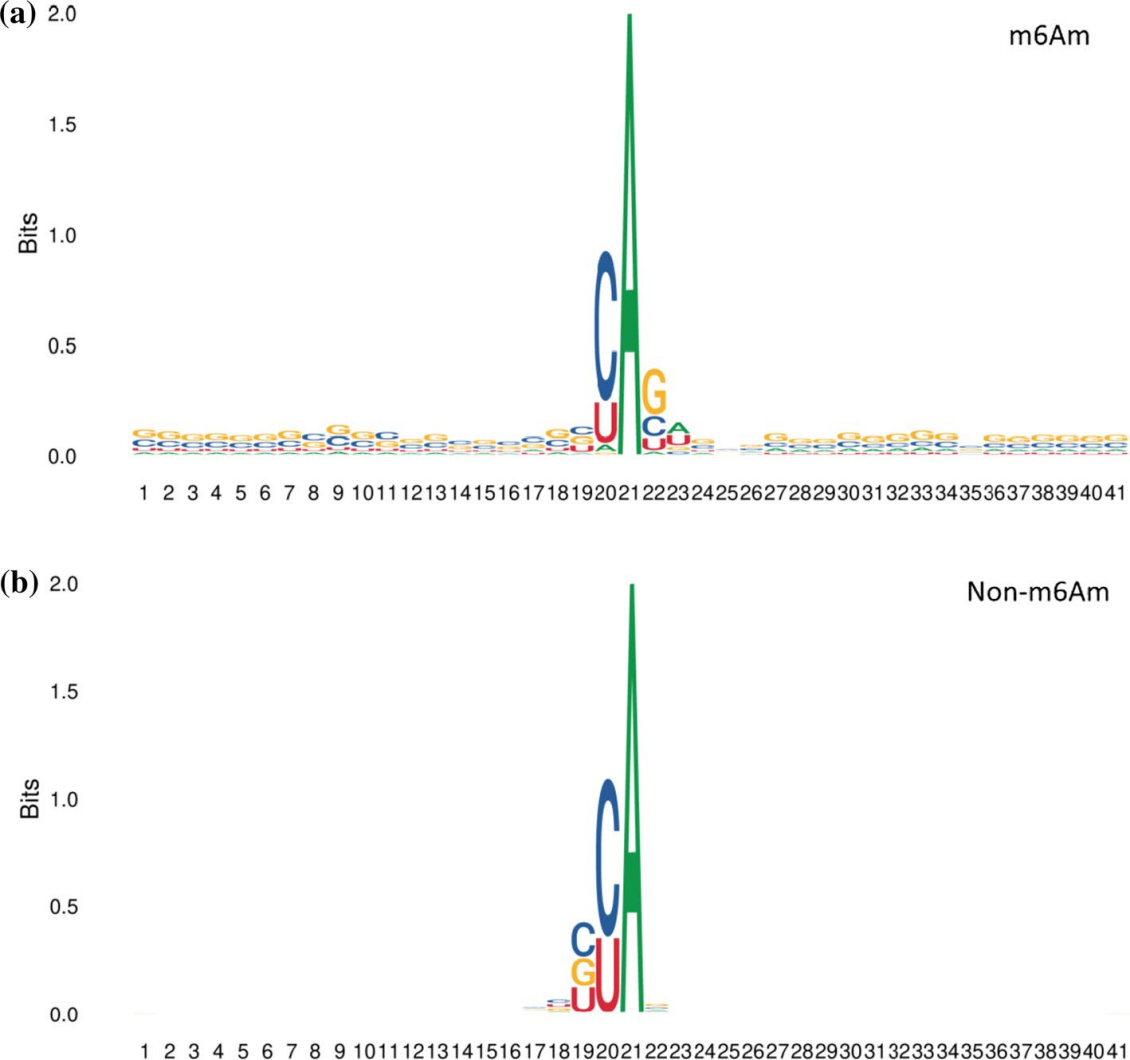
A two-sample logo of m^6^Am sites and Non-m^6^Am sites with L = 41

In this study, we explored the frequency of occurrence of 40 nucleotide bases around the m^6^Am site on RNA sequences to find potential consensus motifs for the sequences. We used an efficient tool, TWO Sample Logo [45], to discover position-specific sign composition differences at the m^6^Am site. In this study, adenine (A) is at the center of the RNA sequence fragment with 20 nucleotides both before and after it. The experimental results were shown in Fig. 10. Nucleotides such as C(cytosine) and U(uracil) were highly represented near both m^6^Am site and non-m^6^Am site, and both appeared in the left position, which was common to both. However, the frequencies and positions of nucleotides at other positions of m^6^Am site and non-m^6^Am site were different, for example, G (guanine), C (cytosine) and U (uracil) were in high proportions near the right side of m^6^Am site. The frequencies of each nucleotide at the front end of the sequence and the back end of the sequence of non-m^6^Am site were similar to the extent that their expression was not obvious. Such analysis indicated that the distances and frequencies between different nucleotides in the sequence played a crucial role in distinguishing m^6^Am sites from non-m^6^Am sites.

**Fig. 11:**
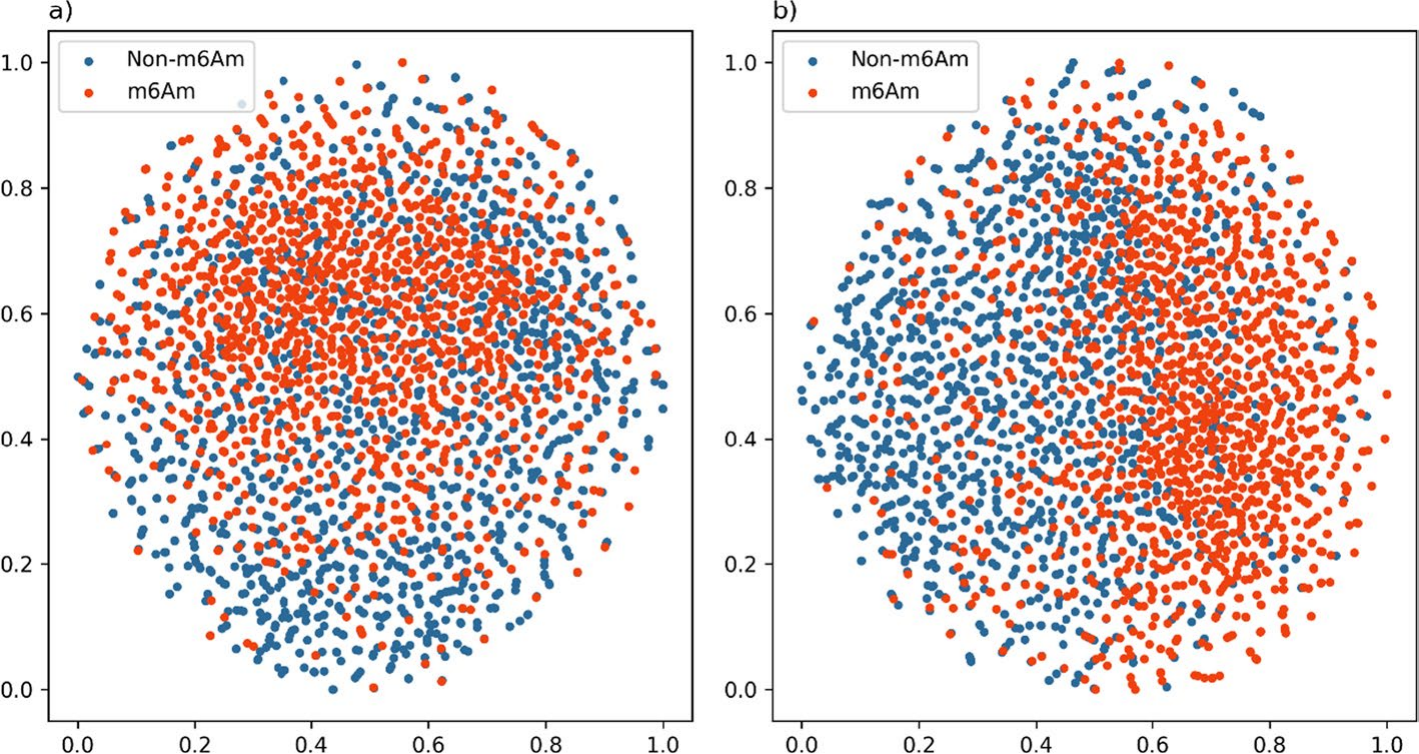
**a** Two-dimensional t-SNE visualization of training data with one-hot encoding. **b** Two-dimensional t-SNE visualization of the learned features from training data by EMDL_m6Am

In addition, we used t-distributed stochastic neighbor embedding (t-SNE) to visualize the two data features. One wasthe projection of the features by one-hot coding of the original training data (Fig. 11a); The other was the total feature projection into two-dimensional space after stitching the important features learned by EMDL_m6Am (3 dimensions) and the original training data one hot encoded features (41*5 dimensions) (Fig. 11b). As can be seen from the Fig. 11, when no prediction is made using EMDL_m6Am, it is not possible to distinguish between positive and negative samples. Conversely, the separation boundary between m^6^Am sites (red data points) and non-m^6^Am sites (blue data points) is very clear after extracting significant features using the three models, which indicates that the proposed EMDL_m6Am has high predictive performance.

## Conclusions

M^6^Am plays a key role in the regulation of RNA and in the identification and treatment of obesity genes and some cancers, therefore, it is crucial to develop predictors that can help detect m^6^Am sites. In this work, we proposed a new predictor, EMDL_m6Am, by stacking three deep learning models together to perform m^6^Am site detection in RNA sequences. It has several advantages over previous studies such as (1) EMDL_m6Am does not need to contain a complex feature extraction process like traditional machine learning, it feature coding is simple and advanced feature extraction is done by the model framework. (2) Traditional CNN cannot extract complex features, in this study, we take an ensemble approach by stacking three powerful deep learning models to extract useful features from different dimensions. Also, we compared multiple traditional machine learning models, advanced deep learning models, and different predictors to highlight the predictive performance of EMDL_m6Am.

In addition, EMDL_m6Am may help to predict other more RNA post-modification sites as a way to discover their novel functions. In future studies, it may be possible to eliminate some of the modules with overlapping functions in the three models to simplify the models, or to explore some of the RNA post-modification sites with unspecified functions.

## Data Availability

It is simple to extract the dataset and source code for this work from (https://github.com/13133989982/EMDL-m6Am).

## Abbreviations

m^6^Am: N6,2′-O-methyladenosine
CNN: Convolutional neural network
DenseNet: Dense convolutional network
DCNN: Inflated convolutional network
MSRN: Deep multiscale residual network
Sn: Sensitivity
Sp: Specificity
ACC: Accuracy
MCC: Mathew correlation coefficient
ROC: Receiver operating characteristics
AUC: Area under ROC curve
PseEIIP: Pseudo-EIIP
NCP: Nucleotide chemical property
ND: Nucleotide density
BiGRU: Bidirectional gating recurrent unit
SENet: Squeeze-and-excitation networks
LSTM: Long short-term memory
BiLSTM: Bidirectional long short-term memory
ReLU: Rectified linear unit
MSRBs: Multi-scale residual blocks
TP: True positive
TN: True negative
FP: False positive
FN: False negative
AUPR: The precision and recall curves
AUROC: The area under the receiver operating characteristic curve
XGBoost: Extreme gradient boosting
t-SNE: T-distributed stochastic neighbor embedding

## References

[1] Boccaletto P, Machnicka MA, Purta E, Piątkowski P, Bagiński B, Wirecki TK (2018). MODOMICS: a database of RNA modification pathways. 2017 update. Nucl Acids Res.

[2] Jonkhout N, Tran J, Smith MA, Schonrock N, Mattick JS, Novoa EM (2017). The RNA modification landscape in human disease. RNA.

[3] Desrosiers R, Friderici K, Rottman F (1974). Identification of methylated nucleosides in messenger RNA from Novikoff hepatoma cells. Proc Natl Acad Sci USA.

[4] Wei C, Gershowitz A, Moss B (1975). N6, O2’-dimethyladenosine a novel methylated ribonucleoside next to the 5’ terminal of animal cell and virus mRNAs. Nature.

[5] Ben-Haim MS, Pinto Y, Moshitch-Moshkovitz S, Hershkovitz V, Kol N, Diamant-Levi T (2021). Dynamic regulation of N6,2′-O-dimethyladenosine (m6Am) in obesity. Nat Commun.

[6] Schwartz S, Mumbach MR, Jovanovic M, Wang T, Maciag K, Bushkin GG (2014). Perturbation of m6A writers reveals two distinct classes of mRNA methylation at internal and 5’ sites. Cell Rep.

[7] Benak D, Kolar F, Zhang L, Devaux Y, Hlavackova M (2023). RNA modification m^6^Am: the role in cardiac biology. Epigenetics.

[8] Cesaro B, Tarullo M, Fatica A (2023). Regulation of Gene Expression by m6Am RNA Modification. Int J Mol Sci.

[9] Fernandez Rodriguez G, Cesaro B, Fatica A (2022). Multiple Roles of m6A RNA Modification in Translational Regulation in Cancer. Int J Mol Sci.

[10] Zhuo W, Sun M, Wang K, Zhang L, Li K, Yi D (2022). m6Am methyltransferase PCIF1 is essential for aggressiveness of gastric cancer cells by inhibiting TM9SF1 mRNA translation. Cell Discov.

[11] Mauer J, Luo X, Blanjoie A, Jiao X, Grozhik AV, Patil DP, et al. Reversible methylation of m6Am in the 5′ cap controls mRNA stability. 2017:43. 10.1038/nature21022 PMC5513158 28002401

[12] Pandey RR, Delfino E, Homolka D, Roithova A, Chen K-M, Li L (2020). The mammalian cap-specific m6Am RNA methyltransferase PCIF1 regulates transcript levels in mouse tissues. Cell Rep.

[13] Boulias K, Toczydłowska-Socha D, Hawley BR, Liberman N, Takashima K, Zaccara S (2019). Identification of the m6Am methyltransferase PCIF1 reveals the location and functions of m6Am in the transcriptome. Mol Cell.

[14] Akichika S, Hirano S, Shichino Y, Suzuki T, Nishimasu H, Ishitani R (2019). Cap-specific terminal N 6-methylation of RNA by an RNA polymerase II-associated methyltransferase. Science.

[15] Hawley BR, Jaffrey SR (2019). Transcriptome-wide mapping of m6 A and m6 Am at single-nucleotide resolution using miCLIP. Curr Protoc Mol Biol.

[16] Koh CWQ, Goh YT, Goh WSS (2019). Atlas of quantitative single-base-resolution N6-methyl-adenine methylomes. Nat Commun.

[17] Sun H, Li K, Zhang X, Liu J, Zhang M, Meng H (2021). m6Am-seq reveals the dynamic m6Am methylation in the human transcriptome. Nat Commun.

[18] Jiang J, Song B, Chen K, Lu Z, Rong R, Zhong Y (2022). m6AmPred: Identifying RNA N6, 2′-O-dimethyladenosine (m6Am) sites based on sequence-derived information. Methods.

[19] Song Z, Huang D, Song B, Chen K, Song Y, Liu G (2021). Attention-based multi-label neural networks for integrated prediction and interpretation of twelve widely occurring RNA modifications. Nat Commun.

[20] Luo Z, Su W, Lou L, Qiu W, Xiao X, Xu Z (2022). DLm6Am: a deep-learning-based tool for identifying N6,2′-O-dimethyladenosine sites in RNA sequences. IJMS.

[21] Wang H, Zhao H, Yan Z, Zhao J, Han J (2021). MDCAN-Lys: a model for predicting succinylation sites based on multilane dense convolutional attention Network. Biomolecules.

[22] Wang H, Yan Z, Liu D, Zhao H, Zhao J (2020). MDC-Kace: a model for predicting lysine acetylation sites based on modular densely connected convolutional networks. IEEE Access.

[23] Niu M, Zou Q, Lin C (2022). CRBPDL: Identification of circRNA-RBP interaction sites using an ensemble neural network approach. PLoS Comput Biol.

[24] Jia J, Wu G, Qiu W (2022). pSuc-FFSEA: predicting lysine succinylation sites in proteins based on feature fusion and stacking ensemble algorithm. Front Cell Dev Biol.

[25] Li W, Godzik A (2006). Cd-hit: a fast program for clustering and comparing large sets of protein or nucleotide sequences. Bioinformatics.

[26] Chen W, Tran H, Liang Z, Lin H, Zhang L (2015). Identification and analysis of the N6-methyladenosine in the Saccharomyces cerevisiae transcriptome. Sci Rep.

[27] Rehman MU, Tayara H, Chong KT. DL-M6A: identification of N6-methyladenosine sites in mammals using deep learning based on different encoding schemes. IEEE/ACM Trans Comput Biol Bioinform. 2022. 10.1109/TCBB.2022.3192572 35857733

[28] Bari ATMG, Reaz MR, Choi H-J, Jeong B-S, Hong B, Meng X, Chen L, Winiwarter W, Song W (2013). DNA encoding for splice site prediction in large DNA sequence. Database systems for advanced applications.

[29] Yang H, Lv H, Ding H, Chen W, Lin H (2018). iRNA-2OM: a sequence-based predictor for Identifying 2’-O-Methylation sites in homo sapiens. J Comput Biol.

[30] Chen W, Feng P, Tang H, Ding H, Lin H (2016). RAMPred: identifying the N1-methyladenosine sites in eukaryotic transcriptomes. Sci Rep.

[31] Chen W, Tang H, Lin H (2017). MethyRNA: a web server for identification of N6-methyladenosine sites. J Biomol Struct Dyn.

[32] Huang G, Liu Z, van der Maaten L, Weinberger KQ. Densely connected convolutional networks. 2017. p. 4700–8.

[33] Jia J, Wu G, Li M, Qiu W. pSuc-EDBAM: predicting lysine succinylation sites in proteins based on ensemble dense blocks and an attention module. Preprint. In Review; 2022. 10.1186/s12859-022-05001-5 PMC9620660 36316638

[34] Jia J, Sun M, Wu G, Qiu W, Jia J, Sun M (2023). DeepDN_iGlu: prediction of lysine glutarylation sites based on attention residual learning method and DenseNet. MBE.

[35] Holschneider M, Kronland-Martinet R, Morlet J, Tchamitchian Ph, Combes J-M, Grossmann A, Tchamitchian P (1990). A real-time algorithm for signal analysis with the help of the wavelet transform. Wavelets.

[36] Ku T, Yang Q, Zhang H (2021). Multilevel feature fusion dilated convolutional network for semantic segmentation. Int J Adv Rob Syst.

[37] Liu C, Song J, Ogata H, Akutsu T. MSNet-4mC: learning effective multi-scale representations for identifying DNA N4-methylcytosine sites. Bioinformatics. 2022:btac671. 10.1093/bioinformatics/btac671 36205602

[38] Chaabane M, Williams R, Stephens A, Park J. circDeep: deep learning approach for circular RNA classification from other long non-coding RNA. Bioinformatics (Oxford, England). 2019;36. 10.1093/bioinformatics/btz537 PMC6956777 31268128

[39] Kha Q-H, Ho Q-T, Le NQK (2022). Identifying SNARE proteins using an alignment-free method based on multiscan convolutional neural network and PSSM profiles. J Chem Inf Model.

[40] Le NQK, Ho Q-T, Nguyen V-N, Chang J-S (2022). BERT-promoter: an improved sequence-based predictor of DNA promoter using BERT pre-trained model and SHAP feature selection. Comput Biol Chem.

[41] Wang C-Y, Liao H-YM, Wu Y-H, Chen P-Y, Hsieh J-W, Yeh I-H. CSPNet: a new backbone that can enhance learning capability of CNN. 2020. p. 390–1.

[42] Guan Q, Wang Y, Ping B, Li D, Du J, Qin Y (2019). Deep convolutional neural network VGG-16 model for differential diagnosing of papillary thyroid carcinomas in cytological images: a pilot study. J Cancer.

[43] He K, Zhang X, Ren S, Sun J. Deep residual learning for image recognition. 2016. p. 770–8.

[44] Xiao J, Wang J, Cao S, Li B (2020). Application of a novel and improved VGG-19 network in the detection of workers wearing masks. J Phys Conf Ser.

[45] Vacic V, Iakoucheva LM, Radivojac P (2006). Two sample logo: a graphical representation of the differences between two sets of sequence alignments. Bioinformatics.

